# SIRT1: A Novel Protective Molecule in Pre-eclampsia

**DOI:** 10.7150/ijms.73012

**Published:** 2022-05-29

**Authors:** Zhenzhen Liu, Chengjie Wang, Jiangnan Pei, Mingqing Li, Weirong Gu

**Affiliations:** 1 Shanghai Key Laboratory of Female Reproductive Endocrine Related Diseases, Shanghai 200011, China.; 2 Department of Obstetrics and Gynecology, Obstetrics and Gynecology Hospital of Fudan University, Shanghai 200011, China.

**Keywords:** pre-eclampsia (PE), SIRT1, trophoblasts, endothelial cells (ECs), resveratrol

## Abstract

Pre-eclampsia is a severe pregnant complication, mainly characterized by insufficient trophoblast invasion, impaired uterine spiral artery remodeling, placental hypoxia and ischemia, and endothelial dysfunction. However, the potential mechanisms of pre-eclampsia remain unclear. SIRT1 is a NAD+-dependent deacetylase, involving in multiple biological processes, including energy metabolism, oxidative stress, inflammatory response, and cellular autophagy. Several studies showed that SIRT1 might play a vital role in the pathogenesis of pre-eclampsia. In this review, we aim to integrate the latest research on SIRT1 and pre-eclampsia to explore the comprehensive mechanisms of SIRT1 in pre-eclampsia. More specifically, SIRT1 might affect placental development and trophoblast invasion through autophagy and senescence in pre-eclampsia, and SIRT1 protects vascular endothelial cells from oxidative stress, inflammatory response, autophagy, and senescence. Furthermore, SIRT1 deficiency mice showed typical pre-eclampsia-like performances, which can be reversed via direct SIRT1 supplement or SIRT1 agonist treatment. Additionally, resveratrol, a SIRT1 agonist, attenuates vascular endothelial injury and placental dysfunction, and exerts protective effect on decreasing blood pressure. In this review, we provide new insights into the development of pre-eclampsia, which can establish a theoretical basis for prevention and treatment for pre-eclampsia. Besides, we also propose questions that still need to be further addressed in order to elucidate the comprehensive molecular mechanisms of pre-eclampsia in the future.

## Introduction

Pre-eclampsia (PE) is a hypertensive disorder of pregnancy (HDP), characterized by new-onset hypertension and proteinuria at 20-week of pregnancy. It affects 2%-8% pregnancy women worldwide, causing severe fetal and maternal morbidity and mortality 1-3. Although the comprehensive mechanisms of pre-eclampsia remain unknown, the current mainstream view is the two-stage model of disease 4-6. Stage1 mainly manifests as impaired placentation due to inadequate trophoblastic invasion of maternal spiral arteries, which leads to reduced placental perfusion and release of numerous secreted factors causing vascular endothelial dysfunction and multiorgan failure, which is called stage2. Recently, the effects of SIRT1 on the biological functions of trophoblasts and endothelial cells have gradually emerged, and the expression of SIRT1 is lower in serum samples and placental tissues of pre-eclampsia patients. Therefore, we inferred that SIRT1 might play a significant role in the pathogenesis of pre-eclampsia.

SIRT1, a NAD+-dependent deacetylase, mediates various biological functions including oxidative stress, aging, inflammatory response and autophagy via deacetylating multiple substrates, such as NF-κB (nuclear factor-kappaB), FOXOs (forkhead box O), and PPARγ (peroxisome proliferator-activated receptor γ) 7-11. For example, it is reported that SIRT1 promotes the deacetylation of Nrf2 (nuclear factor-erythroid 2 (NF-E2)-related factor2), and increases its transcriptional activity, thereby promoting the expression of downstream two-phase detoxification NQO1 (NADPH quinone oxidoreductase 1) and HO-1 (heme oxygenase-1), and exerting anti-oxidative stress effect in vascular endothelial cells 12-15. In addition, SIRT1 deacetylates and activates eNOs (neuronal nitricoxide synthase) to produce more nitric oxide (NO), which can dilate blood vessels 16. In recent years, the research of SIRT1 in pre-eclampsia has progressed. SIRT1 deficiency attenuates the invasion, migration and proliferation of trophoblasts, thereby participating in the development of pre-eclampsia. Our recent study showed that SIRT1 knockdown mice exhibited significantly pre-eclampsia-like symptoms, suggesting that SIRT1 might be a novel protective biomarker in pre-eclampsia 17.

In this review, we mainly explored the role of SIRT1 in pre-eclampsia from the following four aspects. 1) SIRT1 affects the biological functions of trophoblasts; 2) SIRT1 protects vascular endothelial cells; 3) SIRT1 attenuates the performances of pre-eclampsia in animal models; 4) the effect of SIRT1 agonist resveratrol in pre-eclampsia.

## 2. SIRT1 affects the biological functions of trophoblasts

### 2.1. SIRT1 affects placental development and differentiation

**Figure 1:**
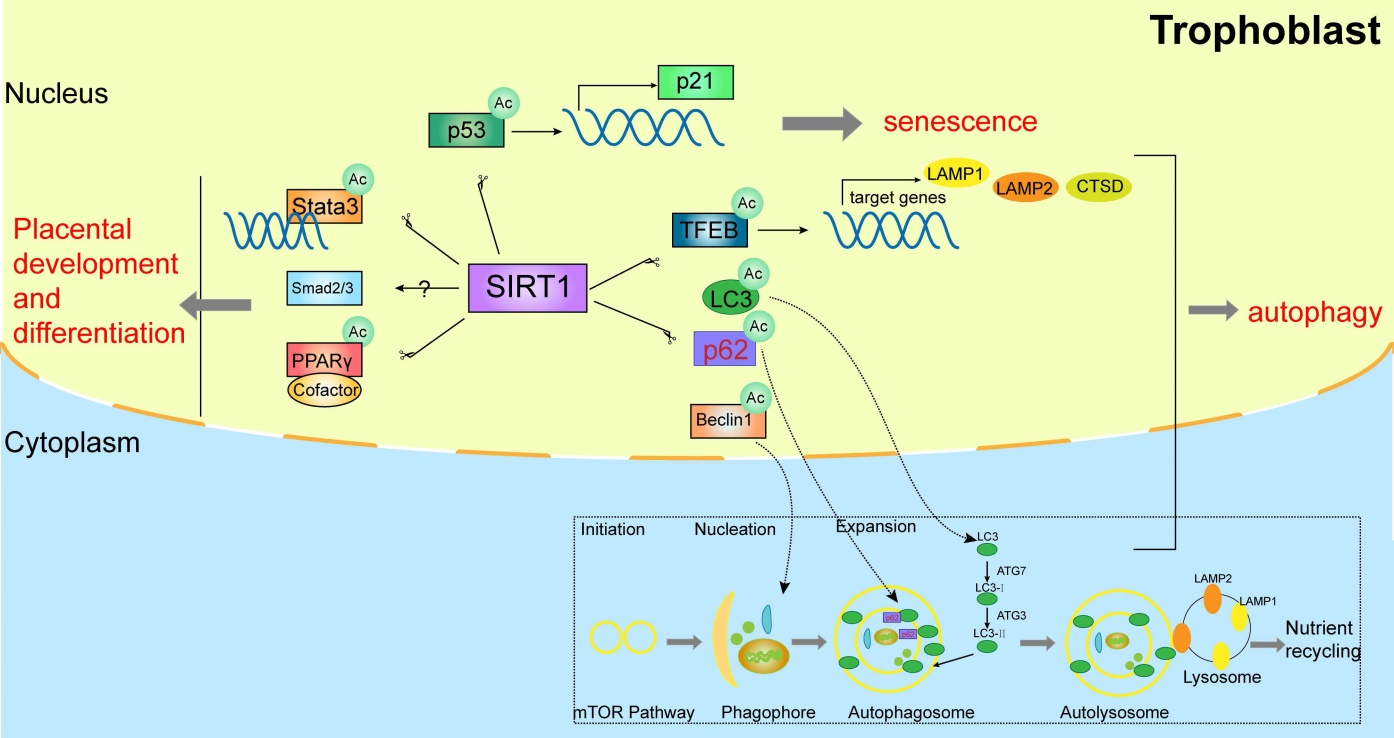
SIRT1 affects the biological functions of trophoblasts.

Trophoblastic dysfunction is a typical feature of pre-eclampsia, resulting in uterine spiral artery remodeling disorder. It is reported that SIRT1 is critical in trophoblast differentiation and placental development 18-21. SIRT1 is lower in placentas and serum samples of pre-eclampsia patients, and is mainly expressed in the nuclei of trophoblasts including syncytiotrophoblasts and cytotrophoblasts in placental tissues 22, 23. SIRT1 possibly involves in trophoblastic maintenance and differentiation by mediating SMAD2/3, STAT3 or PPARγ pathways 24-27. Arul Nambi Rajan et al. 22 found that placentas of SIRT1-null mice were small and showed abnormalities in both labyrinthine layer and junctional zone, and SIRT1-null trophoblast stem cell (TSC) showed blunted differentiation. Specifically, the RNA levels of PPARγ were decreased, and the protein levels of SMAD2, SMAD3 and STAT3 were downregulated in differentiated SIRT1-null TSC. Studies reported that STAT3 was associated with the differentiation of trophoblast giant cells and syncytiotrophoblasts and might be deacetylated and inhibited by SIRT1 25, 28, 29. Additionally, the potential role of PPARγ in trophoblast differentiation and placental development is also highlighted 24, 30, and the activity of PPARγ can be deacetylated and regulated by SIRT1 through recruiting cofactors, such as NCoR1 (nuclear receptor corepressor 1), SMRT (silencing mediator of retinoic acid and thyroid hormone) and Prdm16 (PR domain-containing protein 16) 31, 32. The above-mentioned pathways are shown in Figure 1. Furthermore, our previous research also demonstrated that the placental labyrinthine layer was significantly narrow in *SIRT1^+/-^* mice and the invasive ability was relatively lower in *SIRT1* knockdown trophoblasts 17. This evidence indicated that SIRT1 plays a significant role in placental development and differentiation.

### 2.2. SIRT1 affects trophoblast autophagy

Autophagy is a cellular homeostasis pathway targeted aggregated proteins and damaged organelles for lysosomal degradation 33-37. Importantly, autophagy protects the placentas against pathogens and stress. There are impaired trophoblast autophagy and increased protein accumulation in the placentas of patients with pre-eclampsia 38. Studies showed that SIRT1 prevents H_2_O_2_-induced oxidative stress and apoptosis by mediating autophagy in trophoblasts 39. Mechanistically, some evidence on the autophagic machinery demonstrated that SIRT1 participates in autophagy via deacetylating TFEB (transcription factor EB), LC3 (microtubule associated protein 1 light chain 3), Beclin-1, p62, ATG5 (autophagy-related gene 5), ATG7 (autophagy-related gene 7), and ATG8 (autophagy-related gene 8) in a NAD+-dependent manner 40, 41.

Autophagy-lysosomal biogenesis is tightly regulated by TFEB, which can be deacetylated by SIRT1 and activate the expression of several downstream autophagy-associated genes, such as LAMP1 (lysosomal associated membrane protein 1), LAMP2 (lysosomal associated membrane protein 2) and CTSD (cathepsin D) 42, 43. Furthermore, the initial stage markers of autophagy activation in pre-eclampsia, such as LC3-II, Beclin-1, and SQSTM1 (sequestosome 1) 44-46, were also significantly altered and could be regulated by SIRT1^47^-^49^. The above-mentioned pathways are shown in Figure 1. This evidence demonstrated that SIRT1 might exert a potential role in trophoblastic autophagy by deacetylating multiple substrates.

### 2.3. SIRT1 affects placental senescence

Premature placental senescence is a critical characteristic of pre-eclampsia, with senescence-associated secretory phenotype and increased expression of p53 and p21, which are markers of cellular senescence. SIRT1 is also a specific marker of senescence, and SIRT1 deficiency leads to premature senescence of placentas during placentation 50-53. Interestingly, Xiong et al. found that SIRT1 deficiency promotes the acetylation of P53, elevates the expression level of P21, and impairs trophoblast invasion and migration in advanced maternal age (AMA) pregnancy women, indicating that SIRT1 might involve in the pathogenesis of pre-eclampsia by inducing placental senescence 50.

### 2.4. The functions of other sirtuins proteins in trophoblasts

There are seven orthologs (SIRT1-7) of sirtuins family in mammals 54. All sirtuins deacetylate multiple target proteins using NAD+ as co-substrate and participate in cellular oxidative stress, energy metabolism, and inflammatory response and so on 55. Several reports revealed that sirtuins play a significant role in the development and differentiation of trophoblasts, as shown in Table 1. SIRT2, one member of the sirtuins family, localizes in placental syncytiotrophoblasts and is downregulated in the placentas of patients with pre-eclampsia. It could inhibit proliferation, migration and invasion, and induce necrosis of placental trophoblast cells 56, 57. Additionally, it is reported that SIRT3 affects the migration, invasion, tube formation and necroptosis of trophoblasts and is implicated in the pathogenesis of pre-eclampsia 58. Furthermore, studies showed that SIRT4 might trigger senescence of trophoblasts 59-61. This evidence further confirms our hypothesis that SIRT1 might participate in the pathogenesis of pre-eclampsia by regulating trophoblastic invasion, migration and proliferation.

## 3. SIRT1 protects vascular endothelial cells

**Figure 2:**
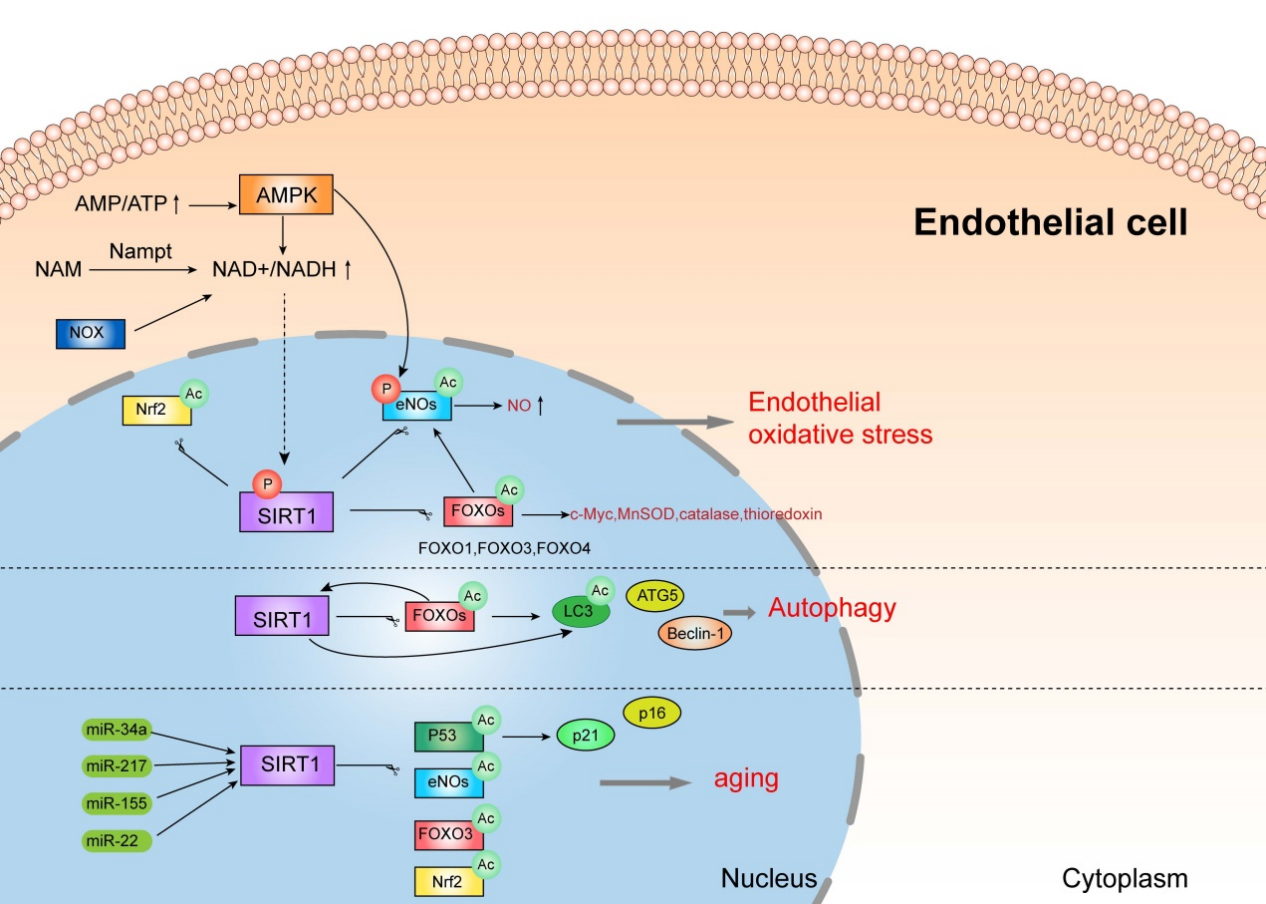
SIRT1 protects vascular endothelial cells.

The dysfunction of endothelial cells is one of the typical features in pre-eclampsia, causing by multiple factors, including oxidative stress, inflammatory response and autophagy and so on. SIRT1, a member of sirtuins family, exerts anti-oxidant, anti-inflammatory, and anti-aging effect. Some research showed that SIRT1 expression is lower in serum samples of pre-eclampsia women, and also decreased in human umbilical vein endothelial cells (HUVECs) incubated with pre-eclamptic serum 63. It is reported that SIRT1 can protect HUVECs from death in pre-eclampsia patients, therefore blocking the development of pre-eclampsia 64. Mechanistically, SIRT1 might protect endothelial cells from oxidative stress, inflammatory response, senescence and autophagy by various pathways, as shown in Figure 2.

### 3.1. SIRT1 protects vascular endothelial cells from oxidative stress and inflammatory response

Oxidative stress and inflammation are closely related pathophysiological process and are both involved in the pathogenesis of pre-eclampsia. Oxidative stress is manifested as an overload of reactive oxygen species (ROS), which always result in inflammatory response and endothelial dysfunction. In pre-eclampsia, mitochondrial function is destroyed and reactive oxygen species (ROS, mainly superoxide anions) are excessively produced, triggering oxidative stress and systemic inflammation 65-68. In vitro model of PE, inhibition of SIRT1 decreases antioxidant activity, and lowers the level of intracellular NO and supernatant nitrite 69, 70. Additionally, SIRT1 also acts as a necessary role in antagonizing oxidative stress and inflammation in the pathogenesis of diabetic vasculopathy 71-73, which is also a critical etiological factor for pre-eclampsia. For instance, the downregulation of SIRT1 induced by hyperglycemia causes vascular dysfunction, while upregulation of SIRT1 attenuates oxidative stress-induced endothelial senescence in diabetic mice 74, 75.

Notably, SIRT1 attenuates oxidative stress and inflammation to regulate vascular endothelial functions through several important signal mediators, such as AMPK, NOXs, eNOs, and FOXOs 76. There is a complex crosstalk network between AMPK and SIRT1. Studies showed that SIRT1 can stimulate AMPK via the modulation of upstream AMPK kinase such as liver kinase B1(LKB1) 76, 77, suppressing the production of ROS and inflammation response in HUVECs, while AMPK influences SIRT1 deacetylation activity by increasing cellular NAD+ levels or directly phosphorylating SIRT1. Furthermore, increased activity of NOX (NADPH oxidase) may also enhance NAD+ content to elevate SIRT1 levels in endothelial cells 78. In addition, SIRT1 deacetylates FOXOs and stimulates FOXO-dependent antioxidant [such as catalase (CAT), manganese superoxide dismutase (MnSOD) and thioredoxin] expression to eliminate ROS in endothelial cells, and prevent endothelial dysfunction 78-80. It is documented that the activation of SIRT1 stimulates the expression of c-Myc by promoting the degradation of FOXO1 to prevent endothelial cell dysfunction and angiogenesis induced by hyperglycemia 81. eNOs, a member of NOS families, is expressed in vascular smooth muscle 82. eNOS plays a crucial role in the pathogenesis of pre-eclampsia, since it makes a great contribution to fight against oxidative stress by producing NO and inhibiting the generation of ROS 83. SIRT1 can directly deacetylate or phosphorylate eNOs, or indirectly stimulate eNOs activity by FOXOs and AMPK pathway 84, which might participate in the pathogenesis of pre-eclampsia. This evidence demonstrated that SIRT1 might protect endothelial cells from oxidative stress and inflammation by interacting with various substrates, which might be associated with pre-eclampsia.

### 3.2. SIRT1 can also protect endothelial cells by autophagy

In endothelial cells, autophagy is mainly regulated by SIRT1/FOXO1 pathway, which might play a crucial role in the pathogenesis of pre-eclampsia 85. Studies showed that SIRT1 actives FOXO1 to protect vascular endothelial cells by regulating autophagy 86. More specifically, SIRT1 deacetylates and activates FOXO1, while FOXO1 can also positively regulate the expression of SIRT1 after activation 87. FOXO1 is closely related to autophagy, since FOXO1 modulates the expression of many autophagy related proteins such as LC3, ATG5 and Beclin-1^88^. These results suggested that SIRT1 protects vascular endothelial cells by regulating autophagy via many pathways.

### 3.3. SIRT1 can also protect endothelial cells from senescence

Vascular endothelial senescence is a major risk factor for cardiovascular disease and a leading cause of death in patients 89, 90. Interestingly, patients with pre-eclampsia exhibit senescence and dysfunction of endothelial progenitor cells 91, 92. And SIRT1 protects endothelial cells from senescence by various pathways, such as p53, eNOs, Nrf2, FOXO3, and p21/p16, which can be regulated by several miRNA, including miR-217, miR-34a, miR-155, and miR-22 93-99. However, more evidence is needed to further verify the functions of SIRT1 in endothelial aging.

This evidence demonstrated that SIRT1 can protect endothelial cells from oxidative stress, inflammatory response, senescence and autophagy by deacetylating various substrates, which might be involved in the pathogenesis of pre-eclampsia.

## 4. SIRT1 attenuates the performances of pre-eclampsia in animal models

### 4.1. SIRT1 knockdown drives the development of pre-eclampsia

Studies reported that SIRT1 is decreased in placentas and serum samples of pre-eclampsia patients, as well as in placentas of pre-eclampsia mice model 100. Importantly, in our previous research, we found that *SIRT1* knockdown mice (*SIRT1^+/-^* mice) exhibits significant pre-eclampsia-like performances, such as hypertension, proteinuria, fetal growth restriction, kidney injury, and narrow labyrinthine layer, while the manifestations could be reversed after intraperitoneally injecting SRT2104, which is a highly selective agonist of SIRT1^17^. Similarly, Arul Nambi Rajan et al. 22 also found that embryos and placentas were smaller in SIRT1 absence mice, with placentas showing abnormalities in both the labyrinthine layer and junctional zone. Additionally, SIRT1 deficiency mice show multiple developmental defects, ranging from embryonic lethality to postnatal lethality during embryogenesis, with embryo growth restriction 1, 101-103. Furthermore, placentas of SIRT1-KO mice exhibit senescence markers and morphological disruption 50, which is closely associated with the development of pre-eclampsia.

### 4.2. Supplement of SIRT1 attenuates the performances of pre-eclampsia

Recently, Huang et al. found that supplement with SIRT1 recombinant protein improved the blood pressure, angiogenic imbalance, inflammation, and pregnancy outcome in RUPP pre-eclampsia rat model 8. Interestingly, in our previous research, the pre-eclampsia-like performances were reversed after intraperitoneally injecting SRT2104 that can elevate SIRT1 protein expression 17. However, more animal experiments and clinical trials are needed to further verify the role of SIRT1 in pre-eclampsia.

## 5. The effect of SIRT1 agonist resveratrol in pre-eclampsia

Resveratrol (3,5,4´-trihydoxy-trans-stilbene, RESV) is a plant polyphenol found in grape skins and red wine, and mainly functions as SIRT1 agonist. Studies have shown that resveratrol involves in various biological processes, such as anti-oxidation, anti-inflammation, anti-aging and anti-cancer 104. And resveratrol is considered in the treatment of pre-eclampsia according to various pre-clinical experiments and clinical trial.

### 5.1. The effect of resveratrol on trophoblasts or endothelial cells—in vitro experiments

**Figure 3:**
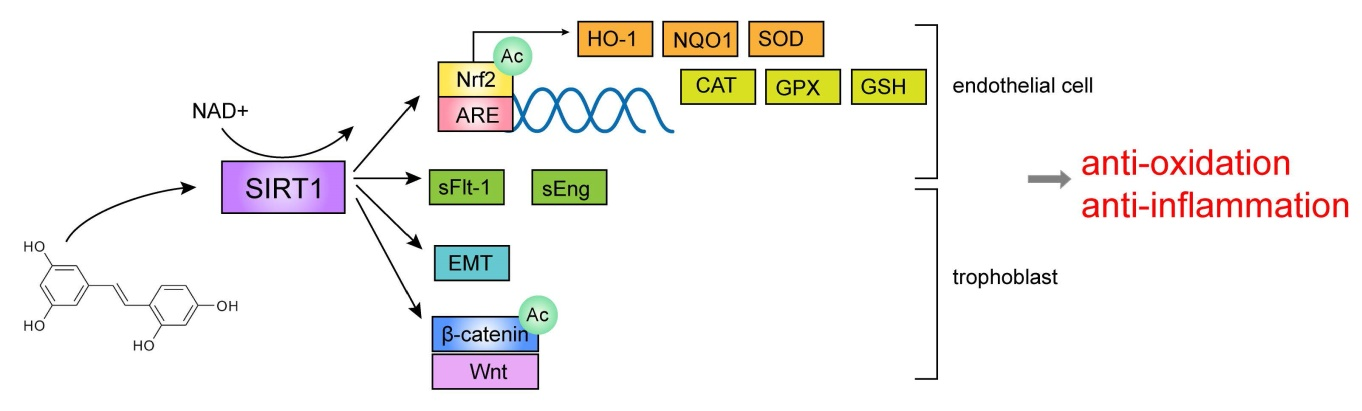
The effect of SIRT1 agonist resveratrol in pre-eclampsia.

Some studies suggested that resveratrol has an anti-hypertensive effect, which is mainly related to inhibiting the release of sFlt-1 (soluble fms-like tyrosine kinase-1) and sEng (soluble endoglin), reducing the expression of pro-inflammatory molecules, and increasing the expression of anti-oxidant molecules. Resveratrol reduced sFlt-1 and sEng secretion from primary trophoblasts and HUVECs 105, 106, and the elevation of sFlt-1 and sEng is an important feature of pre-eclampsia. Additionally, it is reported that resveratrol could reduce oxidative stress by improving some anti-oxidant markers in endothelial cells of pre-eclampsia, including HO-1, NQO1, Nrf2, GSH (glutathione), SOD (superoxide dismutase) and ARE (antioxidant responsive element) 39, 107-109, which are all crucial molecules regulated by SIRT1. Nrf2, a redox-sensitive transcription factor, can be deacetylated and activated by SIRT1 and promotes the genes transcription of downstream detoxification enzymes and antioxidant enzymes 110, 111, such as SOD and HO-1^112^-^114^. In addition, Nrf2 can combine with specific DNA sequence ARE to stimulate the transcription of downstream target genes and antioxidant genes including CAT, SOD, and GPX (glutathione peroxidase) 115. Therefore, resveratrol may play an antioxidant role by upregulating the expression level of SIRT1, thereby activating downstream antioxidant molecules. In addition, some research also reported that resveratrol might promote trophoblast invasion, migration and tube formation by activating epithelial-mesenchymal transition (EMT) and Wnt/β-catenin pathway in pre-eclampsia 116. The above-mentioned pathways are shown in Figure 3. These reports demonstrated that resveratrol, as an agonist of SIRT1, can regulate the functions of trophoblasts and endothelial cells in vitro.

### 5.2. The effect of resveratrol on blood pressure in animal model of pre-eclampsia—in vivo experiments

Interestingly, it is reported that resveratrol can alleviate the symptoms of pre-eclampsia in animal models. Poudel et al. 117 showed that resveratrol improves artery blood flow and increases fetal weight in *COMT^-/-^* mice but not in *eNOS^-/-^* mice, which are both animal models of pre-eclampsia. Furthermore, resveratrol reverses the blood pressure and the concentration of urine protein, and inhibits the oxidative stress in L-NAME-induced pre-eclampsia rat model 109, 116. However, Ozlem's research is different from the results of the above-mentioned studies, possibly due to the differentially experimental methodology 5. Therefore, resveratrol reduces blood pressure in pre-eclampsia animal models, indicating that SIRT1 might modulate the progression of pre-eclampsia.

### 5.3. The effect of resveratrol on blood pressure in pre-eclampsia—clinical trials

Furthermore, several clinical trials also found that resveratrol can decrease blood pressure in hypertensive patients. A randomized clinical trial showed that taking resveratrol can significantly reduce hypertensive symptoms in pre-eclampsia patients, compared with the control group 118. Several meta-analyses and reviews also verify the efficacy of resveratrol in pre-eclampsia 119-122. Moreover, resveratrol also improves flow-mediated dilatation in obese patients and has a controversial anti-hypertensive effect on hypertensive patients 123-127. This evidence suggested that resveratrol might reduce the blood pressure in hypertensive patients, and might play a crucial role in improving the symptoms of pre-eclampsia in a SIRT1 dependent manner. However, resveratrol might also play an anti-hypertensive effect through other pathways, which needs more experiments to verify.

## Discussion

In this review, we systematically concluded the role of SIRT1 in pre-eclampsia. SIRT1 can affect the development, differentiation, autophagy and senescence of trophoblasts, thereby regulating their invasion and migration, and participating in the remodeling process of spiral arteries 22, 39, 50. In addition, SIRT1 can also participate in vascular endothelial dysfunction by mediating inflammatory response, oxidative stress, autophagy and aging, and reverse the progression of pre-eclampsia 69, 70, 86. Interestingly, SIRT1 knockout mice exhibited significant pre-eclampsia-like performances, which can be attenuated by SIRT1 supplementation 8, 17. Moreover, the SIRT1 agonist resveratrol also shows a strong anti-hypertensive effect, and might function by increasing the expression level of SIRT1 protein 109, 116-118. However, since resveratrol can also act in other ways, further validation is needed. This evidence suggests that SIRT1 might be an important marker in the pathogenesis of pre-eclampsia.

However, there are still many problems needed further experimental validation. For example, some studies have found that SIRT1 can regulate trophoblast autophagy, but the regulatory mechanisms are not yet completely definite. In addition, SIRT1 is also an important anti-aging molecule involved in a variety of aging-related diseases 8. However, the specific mechanisms of SIRT1 in placental aging need to be further elucidated. Furthermore, it is not clear whether SIRT1 is involved in the progression of pre-eclampsia through other ways, such as abnormal placental metabolism. A typical example is that lipid abnormalities develop in placentas of pre-eclampsia patients. Recent research showed a possible role for LXRβ (liver X receptors beta) as a transcriptional regulator in pre-eclampsia 128. LXRβ is a key regulator of lipid homeostasis, and can be deacetylated by SIRT1^129^. However, the functions and mechanisms of SIRT1 and LXRβ in pre-eclampsia remain unclear. Moreover, the upstream molecular mechanisms of SIRT1 in pre-eclampsia also needs to be further elucidated. Our previous study found that progesterone can significantly improve the pre-eclampsia-like symptoms in SIRT1 knockdown mice 17, indicating that progesterone might act as an upstream regulator of SIRT1. These issues still need more experiments and clinical trials to further verify, which is also the direction of our future research.

## Figures and Tables

**Table 1:** The effect of sirtuins family in trophoblasts

Source	Sirtuins	Expression	Location	Effect in trophoblasts	Mechanisms
Arul Nambi Rajan et al. Lappas et al. Barak et al. Borg et al. Erlebacher et al. Tang et al.	SIRT1 22-27	Downregulated in placentas and serum samples from PE, significantly lower after adjusting for gestational age (WB, qPCR, IHC)	Placental syncytiotrophoblasts and cytotrophoblasts (IHC)	SIRT1promotes development, differentiation, migration, invasion, and angiogenesis, while inhibits apoptosis, and senescence of trophoblasts. Furthermore, SIRT1 exerts anti-inflammatory effects and anti-oxidative stress in trophoblasts	SMAD2/3, STAT3 or PPARγ pathways; triggering p53 deacetylation; medicating autophagy
Yu et al. Hannan et al.	SIRT2 56, 57	Downregulated in placentas from PE, but no significance after adjusting for gestational age (microarray, WB, qPCR, IHC)	Placental syncytiotrophoblasts, scattered interstitial cells, the endothelial cells lining, and the vessel walls of the placental villi (IHC)	SIRT2 deficiency inhibits proliferation, migration and invasion, while promotes apoptosis and necroptosis of trophoblasts	Triggering p65 deacetylation
Yu et al.	SIRT3 58	Downregulated in placentas from PE, but no significance after adjusting for gestational age (WB, qPCR, IHC)	Placental syncytiotrophoblasts and cytotrophoblasts (IHC)	SIRT3 deficiency inhibits proliferation, migration, invasion and tube formation, while promotes cell death and necroptosis of trophoblasts	——
Castex et al. Sandvoß et al. Bartho et al.	SIRT4 59-61	Upregulated in HUVECs from HELLP, but no difference in placentas of FGR	——	SIRT4 triggers senescence of trophoblasts	Induced by inactivation of LSD1
Lim et al.	SIRT6 62	Downregulated in fetal membranes from preterm labor	Placental chorionic trophoblasts and decidua tissues, fetal membranes, and amnion epithelium	——	——

## Abbreviations

PE: pre-eclampsia
ECs: endothelial cells
SIRT1: sirtuin1
Ac: acetylation
P: phosphorylation
NF-κB: nuclear factor-kappaB
PPARγ: peroxisome proliferator-activated receptor gamma
PGC-1α: peroxisome proliferator-activated receptor-gamma co-activator-1alpha
TFEB: transcription factor EB
LC3: microtubule associated protein 1 light chain 3
LAMP1: lysosomal associated membrane protein 1
LAMP2: lysosomal associated membrane protein 2
CTSD: cathepsin D
ATG3: autophagy-related gene 3
ATG5: autophagy-related gene 5
ATG7: autophagy-related gene 7
ATG8: autophagy-related gene 8
SQSTM1: sequestosome 1
HUVECs: human umbilical vein endothelial cells
ROS: reactive oxygen species
AMPK: AMP-activated protein kinase
LKB1: liver kinase B1
NAM: nicotinamide
Nampt: nicotinamide phosphoribosyltransferase
NOX: NADPH oxidases
Nrf2: nuclear factor-erythroid 2 (NF-E2)-related factor2
eNOs: neuronal nitricoxide synthase
FOXO: forkhead box O
MnSOD: manganese superoxide dismutase
sFlt-1: soluble fms-like tyrosine kinase-1
sEng: soluble endoglin
HO-1: heme oxygenase-1
GSH: glutathione
SOD: superoxide dismutase
ARE: antioxidant responsive element
CAT: catalase
GPX: glutathione peroxidase
NQO1: NADPH quinone oxidoreductase 1
EMT: epithelial-mesenchymal transition
NCoR1: nuclear receptor corepressor 1
SMRT: silencing mediator of retinoic acid and thyroid hormone
Prdm16: PR domain-containing protein 16
NO: nitric oxide
STAT3: activating signal transducers and activators of transcription 3
RUPP: reduced uterine perfusion pressure
L-NAME: NG-Nitro-L-arginine methyl ester
LXRβ: liver X receptors beta
HELLP: hemolysis, elevated liver enzymes, and low platelet count
FGR: fetal growth restriction
LSD1: lysine-specific demethylase 1
WB: western blotting
qPCR: quantitative real-time PCR
IHC: immunohistochemistry
